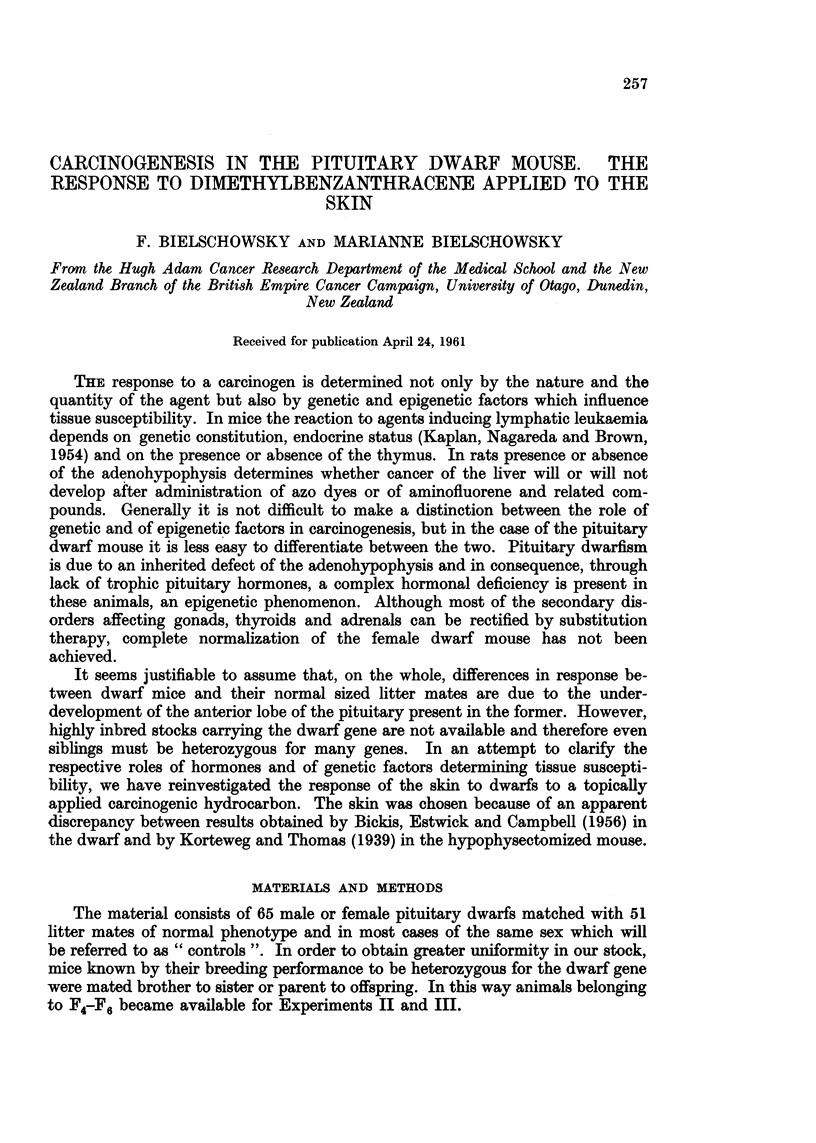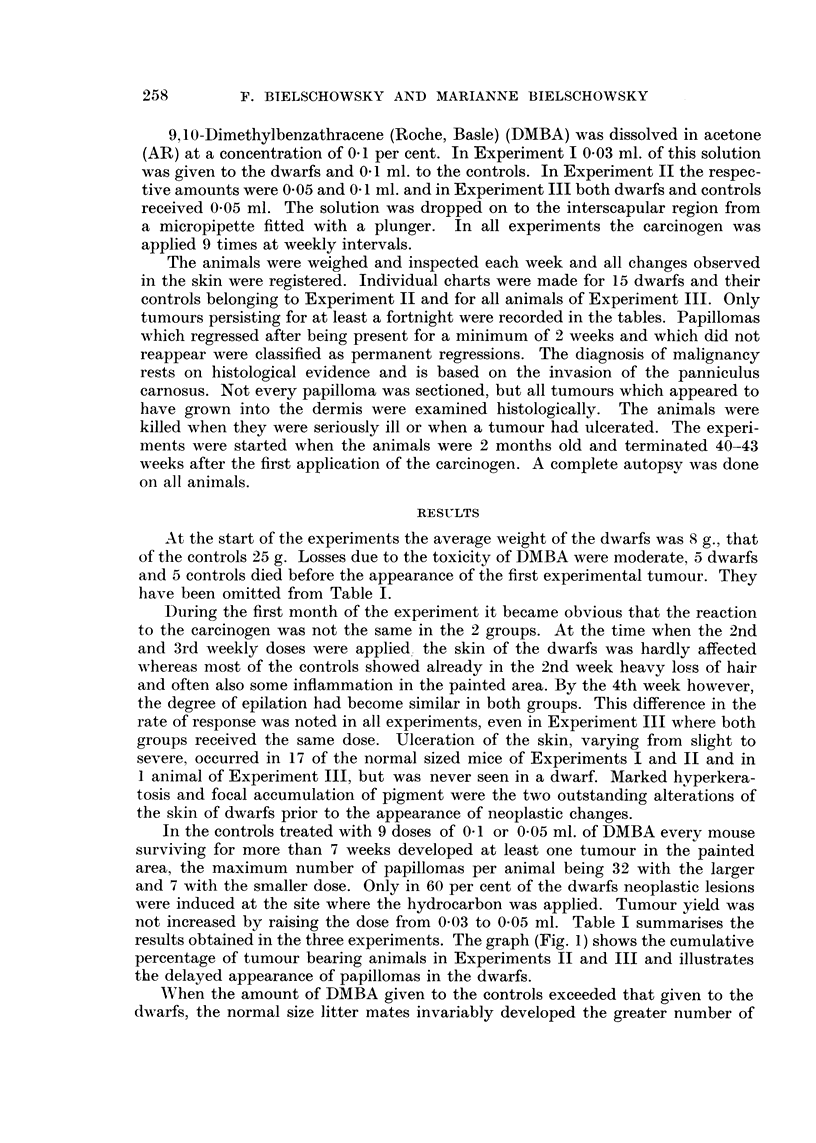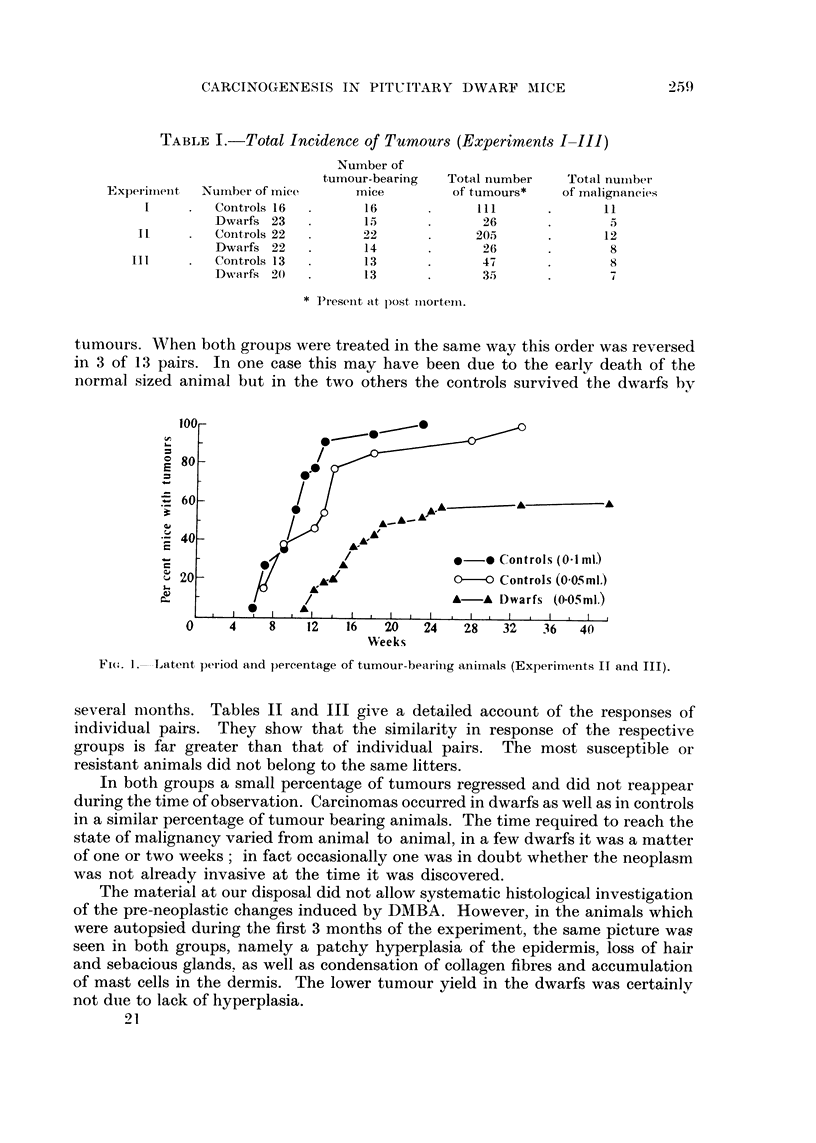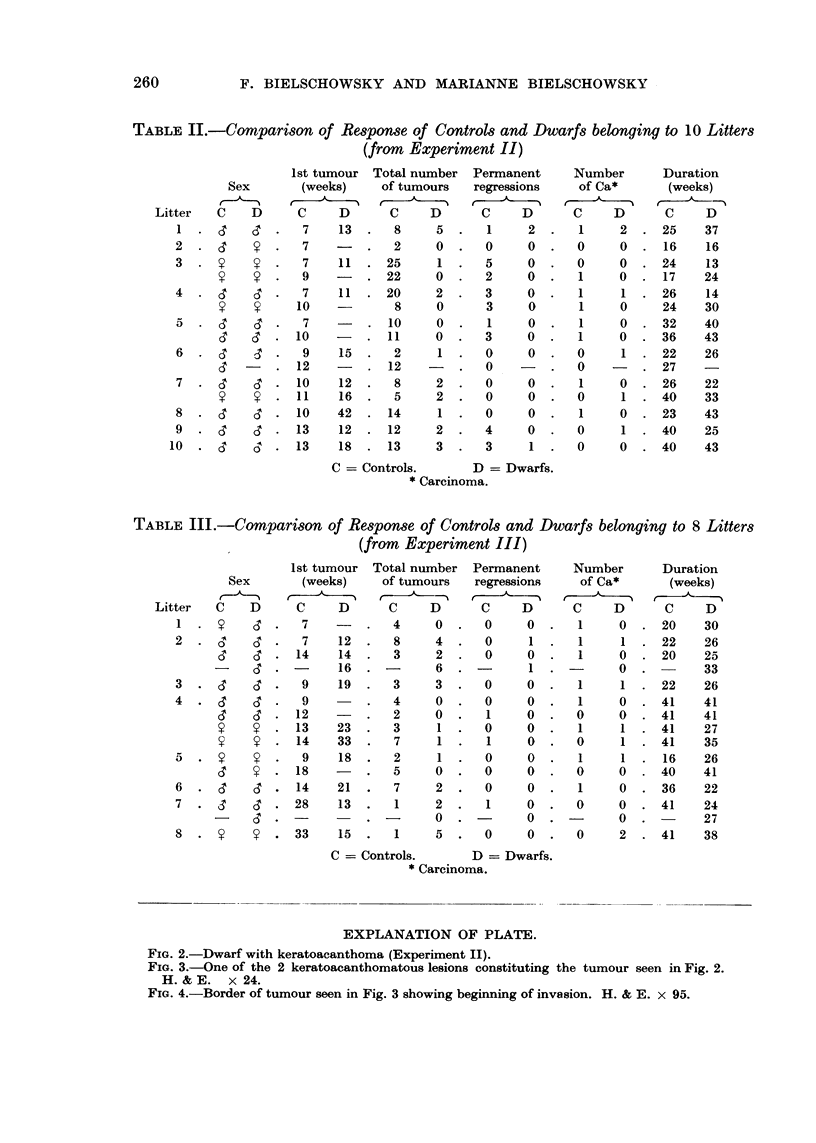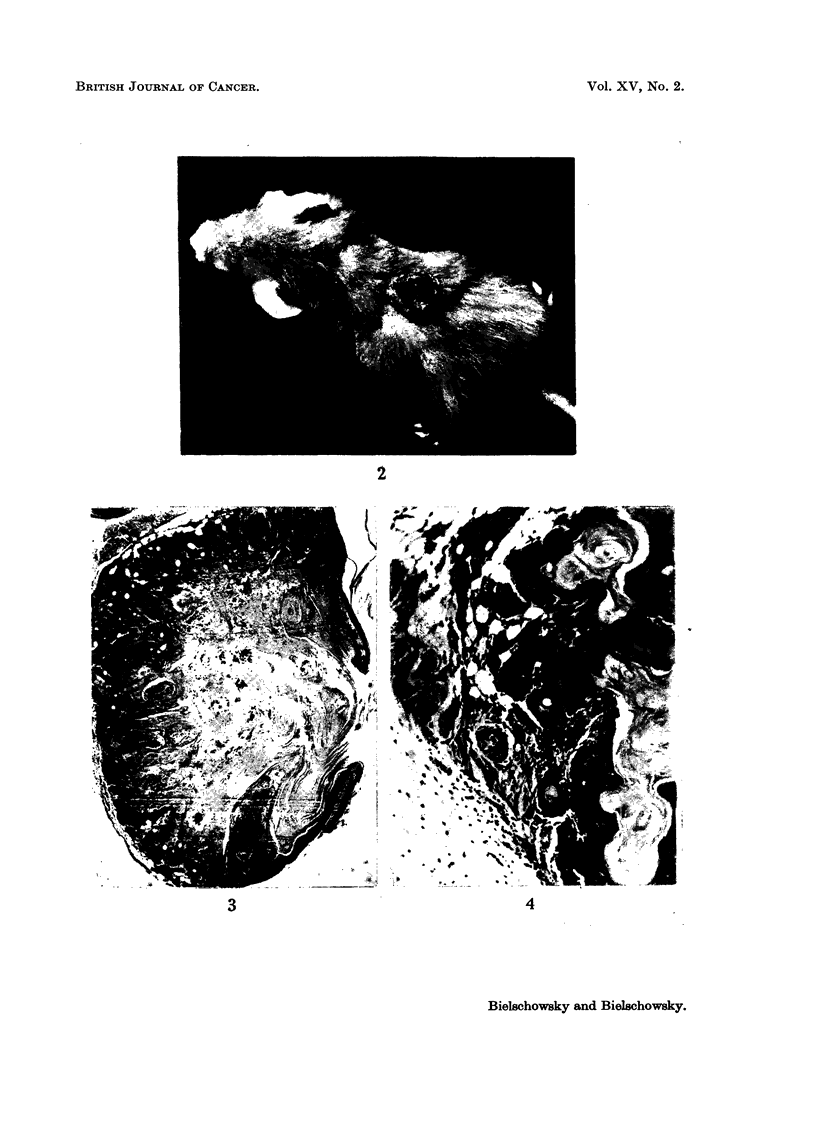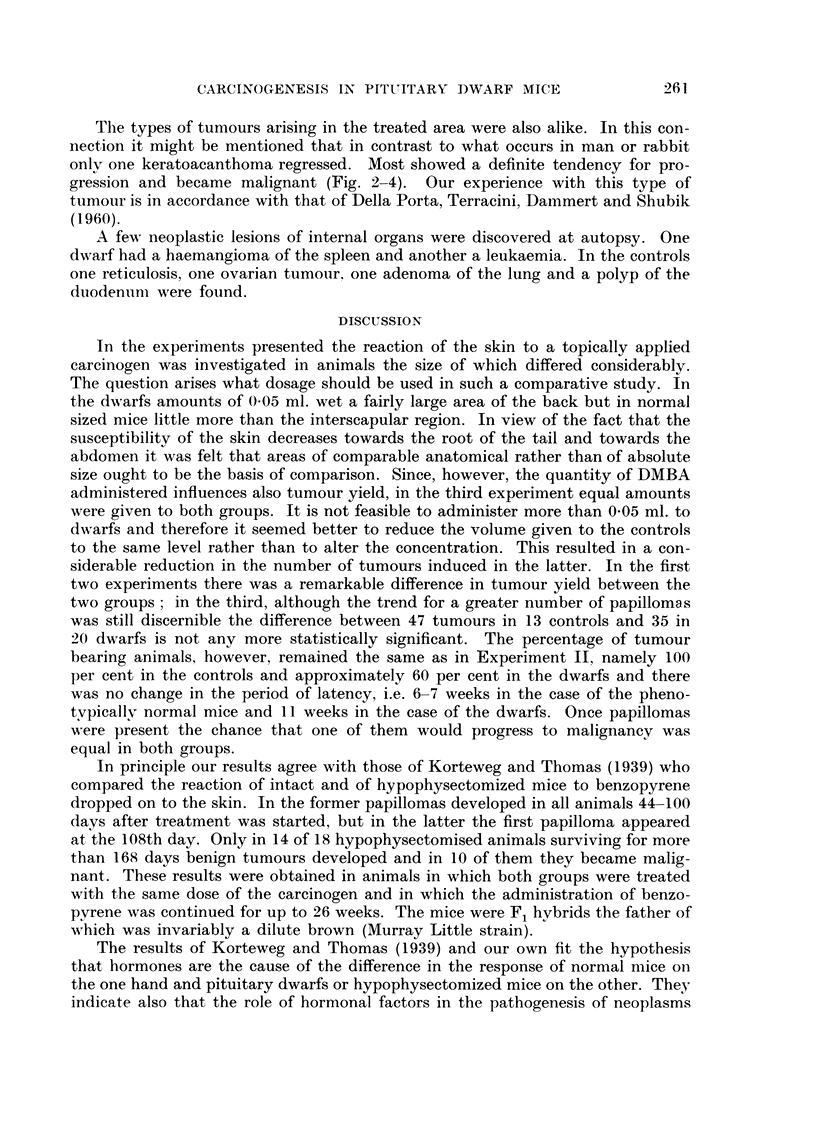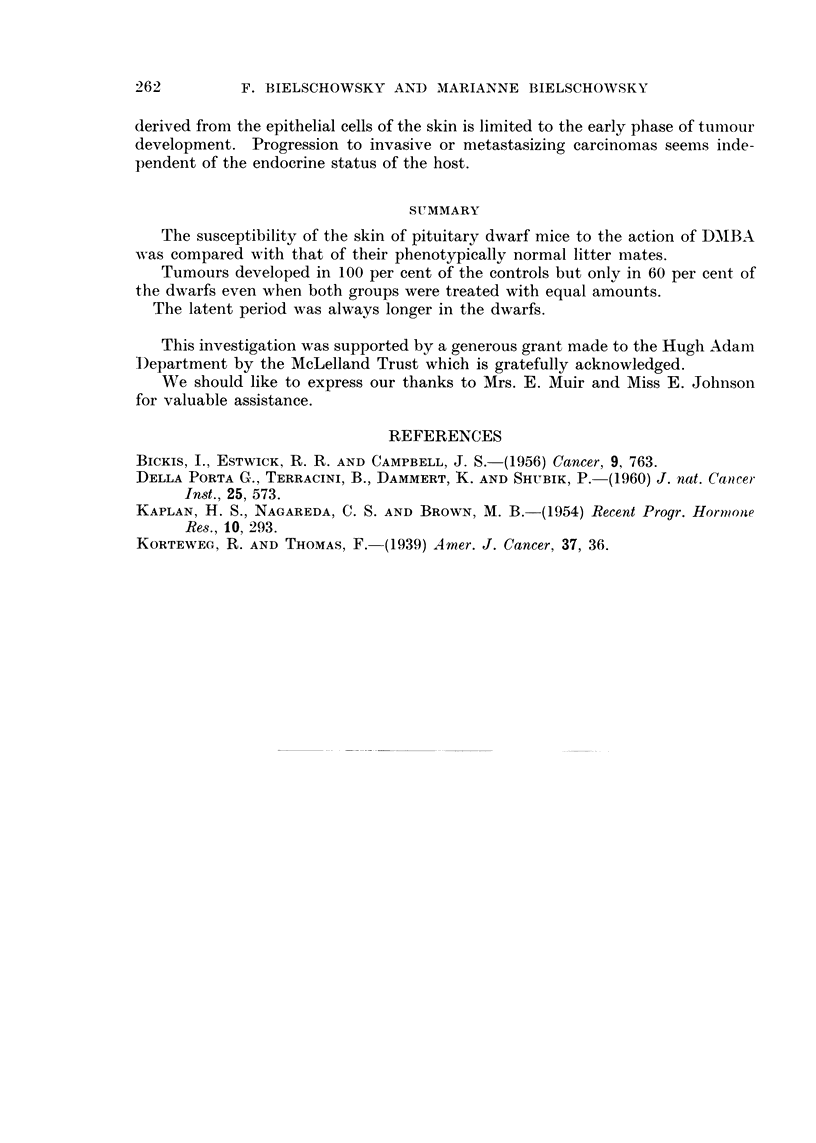# Carcinogenesis in the Pituitary Dwarf Mouse. The Response to Dimethylbenzanthracene Applied to the Skin

**DOI:** 10.1038/bjc.1961.32

**Published:** 1961-06

**Authors:** F. Bielschowsky, Marianne Bielschowsky

## Abstract

**Images:**


### CARCINOGENESIS IN THE PITUITARY DWARF MOUSE. THE RESPONSE TO DIMETHYLBENZANTHRACENE APPLIED TO THE SKIN

F. BIELSCHOWSKY AND MARIANNE BIELSCHOWSKY

*From the Hugh Adam Cancer Research Department of the Medical School and the New Zealand Branch of the British Empire Cancer Campaign, University of Otago, Dunedin, New Zealand*

Received for publication April 24, 1961

THE response to a carcinogen is determined not only by the nature and the quantity of the agent but also by genetic and epigenetic factors which influence tissue susceptibility. In mice the reaction to agents inducing lymphatic leukaemia depends on genetic constitution, endocrine status (Kaplan, Nagareda and Brown, 1954) and on the presence or absence of the thymus. In rats presence or absence of the adenohypophysis determines whether cancer of the liver will or will not develop after administration of azo dyes or of aminofluorene and related compounds. Generally it is not difficult to make a distinction between the role of genetic and of epigenetic factors in carcinogenesis, but in the case of the pituitary dwarf mouse it is less easy to differentiate between the two. Pituitary dwarfism is due to an inherited defect of the adenohypophysis and in consequence, through lack of trophic pituitary hormones, a complex hormonal deficiency is present in these animals, an epigenetic phenomenon. Although most of the secondary disorders affecting gonads, thyroids and adrenals can be rectified by substitution therapy, complete normalization of the female dwarf mouse has not been achieved.

It seems justifiable to assume that, on the whole, differences in response between dwarf mice and their normal sized litter mates are due to the underdevelopment of the anterior lobe of the pituitary present in the former. However, highly inbred stocks carrying the dwarf gene are not available and therefore even siblings must be heterozygous for many genes. In an attempt to clarify the respective roles of hormones and of genetic factors determining tissue susceptibility, we have reinvestigated the response of the skin to dwarfs to a topically applied carcinogenic hydrocarbon. The skin was chosen because of an apparent discrepancy between results obtained by Bickis, Estwick and Campbell (1956) in the dwarf and by Korteweg and Thomas (1939) in the hypophysectomized mouse.

### MATERIALS AND METHODS

The material consists of 65 male or female pituitary dwarfs matched with 51 litter mates of normal phenotype and in most cases of the same sex which will be referred to as " controls ". In order to obtain greater uniformity in our stock, mice known by their breeding performance to be heterozygous for the dwarf gene were mated brother to sister or parent to offspring. In this way animals belonging to $F_4$–$F_6$ became available for Experiments II and III.

9,10-Dimethylbenzathracene (Roche, Basle) (DMBA) was dissolved in acetone (AR) at a concentration of 0·1 per cent. In Experiment I 0·03 ml. of this solution was given to the dwarfs and 0·1 ml. to the controls. In Experiment II the respective amounts were 0·05 and 0·1 ml. and in Experiment III both dwarfs and controls received 0·05 ml. The solution was dropped on to the interscapular region from a micropipette fitted with a plunger. In all experiments the carcinogen was applied 9 times at weekly intervals.

The animals were weighed and inspected each week and all changes observed in the skin were registered. Individual charts were made for 15 dwarfs and their controls belonging to Experiment II and for all animals of Experiment III. Only tumours persisting for at least a fortnight were recorded in the tables. Papillomas which regressed after being present for a minimum of 2 weeks and which did not reappear were classified as permanent regressions. The diagnosis of malignancy rests on histological evidence and is based on the invasion of the panniculus carnosus. Not every papilloma was sectioned, but all tumours which appeared to have grown into the dermis were examined histologically. The animals were killed when they were seriously ill or when a tumour had ulcerated. The experiments were started when the animals were 2 months old and terminated 40–43 weeks after the first application of the carcinogen. A complete autopsy was done on all animals.

### RESULTS

At the start of the experiments the average weight of the dwarfs was 8 g., that of the controls 25 g. Losses due to the toxicity of DMBA were moderate, 5 dwarfs and 5 controls died before the appearance of the first experimental tumour. They have been omitted from Table I.

During the first month of the experiment it became obvious that the reaction to the carcinogen was not the same in the 2 groups. At the time when the 2nd and 3rd weekly doses were applied the skin of the dwarfs was hardly affected whereas most of the controls showed already in the 2nd week heavy loss of hair and often also some inflammation in the painted area. By the 4th week however, the degree of epilation had become similar in both groups. This difference in the rate of response was noted in all experiments, even in Experiment III where both groups received the same dose. Ulceration of the skin, varying from slight to severe, occurred in 17 of the normal sized mice of Experiments I and II and in 1 animal of Experiment III, but was never seen in a dwarf. Marked hyperkeratosis and focal accumulation of pigment were the two outstanding alterations of the skin of dwarfs prior to the appearance of neoplastic changes.

In the controls treated with 9 doses of 0·1 or 0·05 ml. of DMBA every mouse surviving for more than 7 weeks developed at least one tumour in the painted area, the maximum number of papillomas per animal being 32 with the larger and 7 with the smaller dose. Only in 60 per cent of the dwarfs neoplastic lesions were induced at the site where the hydrocarbon was applied. Tumour yield was not increased by raising the dose from 0·03 to 0·05 ml. Table I summarises the results obtained in the three experiments. The graph (Fig. 1) shows the cumulative percentage of tumour bearing animals in Experiments II and III and illustrates the delayed appearance of papillomas in the dwarfs.

When the amount of DMBA given to the controls exceeded that given to the dwarfs, the normal size litter mates invariably developed the greater number of

TABLE I.—*Total Incidence of Tumours (Experiments I–III)*

| Experiment | Number of mice | Number of tumour-bearing mice | Total number of tumours* | Total number of malignancies |
|---|---|---|---|---|
| I | Controls 16 | 16 | 111 | 11 |
| | Dwarfs 23 | 15 | 26 | 5 |
| II | Controls 22 | 22 | 205 | 12 |
| | Dwarfs 22 | 14 | 26 | 8 |
| III | Controls 13 | 13 | 47 | 8 |
| | Dwarfs 20 | 13 | 35 | 7 |

* Present at post mortem.

tumours. When both groups were treated in the same way this order was reversed in 3 of 13 pairs. In one case this may have been due to the early death of the normal sized animal but in the two others the controls survived the dwarfs by

FIG. 1.—Latent period and percentage of tumour-bearing animals (Experiments II and III).

several months. Tables II and III give a detailed account of the responses of individual pairs. They show that the similarity in response of the respective groups is far greater than that of individual pairs. The most susceptible or resistant animals did not belong to the same litters.

In both groups a small percentage of tumours regressed and did not reappear during the time of observation. Carcinomas occurred in dwarfs as well as in controls in a similar percentage of tumour bearing animals. The time required to reach the state of malignancy varied from animal to animal, in a few dwarfs it was a matter of one or two weeks ; in fact occasionally one was in doubt whether the neoplasm was not already invasive at the time it was discovered.

The material at our disposal did not allow systematic histological investigation of the pre-neoplastic changes induced by DMBA. However, in the animals which were autopsied during the first 3 months of the experiment, the same picture was seen in both groups, namely a patchy hyperplasia of the epidermis, loss of hair and sebacious glands, as well as condensation of collagen fibres and accumulation of mast cells in the dermis. The lower tumour yield in the dwarfs was certainly not due to lack of hyperplasia.

TABLE II.—*Comparison of Response of Controls and Dwarfs belonging to* 10 *Litters (from Experiment II)*

| Litter | Sex | | 1st tumour (weeks) | | Total number of tumours | | Permanent regressions | | Number of Ca* | | Duration (weeks) | |
|---|---|---|---|---|---|---|---|---|---|---|---|---|
| | C | D | C | D | C | D | C | D | C | D | C | D |
| 1 | ♂ | ♂ | 7 | 13 | 8 | 5 | 1 | 2 | 1 | 2 | 25 | 37 |
| 2 | ♂ | ♀ | 7 | — | 2 | 0 | 0 | 0 | 0 | 0 | 16 | 16 |
| 3 | ♀ | ♀ | 7 | 11 | 25 | 1 | 5 | 0 | 0 | 0 | 24 | 13 |
| | ♀ | ♀ | 9 | — | 22 | 0 | 2 | 0 | 1 | 0 | 17 | 24 |
| 4 | ♂ | ♂ | 7 | 11 | 20 | 2 | 3 | 0 | 1 | 1 | 26 | 14 |
| | ♀ | ♀ | 10 | — | 8 | 0 | 3 | 0 | 1 | 0 | 24 | 30 |
| 5 | ♂ | ♂ | 7 | — | 10 | 0 | 1 | 0 | 1 | 0 | 32 | 40 |
| | ♂ | ♂ | 10 | — | 11 | 0 | 3 | 0 | 1 | 0 | 36 | 43 |
| 6 | ♂ | ♂ | 9 | 15 | 2 | 1 | 0 | 0 | 0 | 1 | 22 | 26 |
| | ♂ | — | 12 | — | 12 | — | 0 | — | 0 | — | 27 | — |
| 7 | ♂ | ♂ | 10 | 12 | 8 | 2 | 0 | 0 | 1 | 0 | 26 | 22 |
| | ♀ | ♀ | 11 | 16 | 5 | 2 | 0 | 0 | 0 | 1 | 40 | 33 |
| 8 | ♂ | ♂ | 10 | 42 | 14 | 1 | 0 | 0 | 1 | 0 | 23 | 43 |
| 9 | ♂ | ♂ | 13 | 12 | 12 | 2 | 4 | 0 | 0 | 1 | 40 | 25 |
| 10 | ♂ | ♂ | 13 | 18 | 13 | 3 | 3 | 1 | 0 | 0 | 40 | 43 |

C = Controls. D = Dwarfs.
* Carcinoma.

TABLE III.—*Comparison of Response of Controls and Dwarfs belonging to* 8 *Litters (from Experiment III)*

| Litter | Sex | | 1st tumour (weeks) | | Total number of tumours | | Permanent regressions | | Number of Ca* | | Duration (weeks) | |
|---|---|---|---|---|---|---|---|---|---|---|---|---|
| | C | D | C | D | C | D | C | D | C | D | C | D |
| 1 | ♀ | ♂ | 7 | — | 4 | 0 | 0 | 0 | 1 | 0 | 20 | 30 |
| 2 | ♂ | ♂ | 7 | 12 | 8 | 4 | 0 | 1 | 1 | 1 | 22 | 26 |
| | ♂ | ♂ | 14 | 14 | 3 | 2 | 0 | 0 | 1 | 0 | 20 | 25 |
| | — | ♂ | — | 16 | — | 6 | — | 1 | — | 0 | — | 33 |
| 3 | ♂ | ♂ | 9 | 19 | 3 | 3 | 0 | 0 | 1 | 1 | 22 | 26 |
| 4 | ♂ | ♂ | 9 | — | 4 | 0 | 0 | 0 | 1 | 0 | 41 | 41 |
| | ♂ | ♂ | 12 | — | 2 | 0 | 1 | 0 | 0 | 0 | 41 | 41 |
| | ♀ | ♀ | 13 | 23 | 3 | 1 | 0 | 0 | 1 | 1 | 41 | 27 |
| | ♀ | ♀ | 14 | 33 | 7 | 1 | 1 | 0 | 0 | 1 | 41 | 35 |
| 5 | ♀ | ♀ | 9 | 18 | 2 | 1 | 0 | 0 | 1 | 1 | 16 | 26 |
| | ♂ | ♀ | 18 | — | 5 | 0 | 0 | 0 | 0 | 0 | 40 | 41 |
| 6 | ♂ | ♂ | 14 | 21 | 7 | 2 | 0 | 0 | 1 | 0 | 36 | 22 |
| 7 | ♂ | ♂ | 28 | 13 | 1 | 2 | 1 | 0 | 0 | 0 | 41 | 24 |
| | — | ♂ | — | — | — | 0 | — | 0 | — | 0 | — | 27 |
| 8 | ♀ | ♀ | 33 | 15 | 1 | 5 | 0 | 0 | 0 | 2 | 41 | 38 |

C = Controls. D = Dwarfs.
* Carcinoma.

### EXPLANATION OF PLATE.

FIG. 2.—Dwarf with keratoacanthoma (Experiment II).

FIG. 3.—One of the 2 keratoacanthomatous lesions constituting the tumour seen in Fig. 2. H. & E. × 24.

FIG. 4.—Border of tumour seen in Fig. 3 showing beginning of invasion. H. & E. × 95.

2

3

4

The types of tumours arising in the treated area were also alike. In this connection it might be mentioned that in contrast to what occurs in man or rabbit only one keratoacanthoma regressed. Most showed a definite tendency for progression and became malignant (Fig. 2–4). Our experience with this type of tumour is in accordance with that of Della Porta, Terracini, Dammert and Shubik (1960).

A few neoplastic lesions of internal organs were discovered at autopsy. One dwarf had a haemangioma of the spleen and another a leukaemia. In the controls one reticulosis, one ovarian tumour, one adenoma of the lung and a polyp of the duodenum were found.

### DISCUSSION

In the experiments presented the reaction of the skin to a topically applied carcinogen was investigated in animals the size of which differed considerably. The question arises what dosage should be used in such a comparative study. In the dwarfs amounts of 0·05 ml. wet a fairly large area of the back but in normal sized mice little more than the interscapular region. In view of the fact that the susceptibility of the skin decreases towards the root of the tail and towards the abdomen it was felt that areas of comparable anatomical rather than of absolute size ought to be the basis of comparison. Since, however, the quantity of DMBA administered influences also tumour yield, in the third experiment equal amounts were given to both groups. It is not feasible to administer more than 0·05 ml. to dwarfs and therefore it seemed better to reduce the volume given to the controls to the same level rather than to alter the concentration. This resulted in a considerable reduction in the number of tumours induced in the latter. In the first two experiments there was a remarkable difference in tumour yield between the two groups ; in the third, although the trend for a greater number of papillomas was still discernible the difference between 47 tumours in 13 controls and 35 in 20 dwarfs is not any more statistically significant. The percentage of tumour bearing animals, however, remained the same as in Experiment II, namely 100 per cent in the controls and approximately 60 per cent in the dwarfs and there was no change in the period of latency, i.e. 6–7 weeks in the case of the phenotypically normal mice and 11 weeks in the case of the dwarfs. Once papillomas were present the chance that one of them would progress to malignancy was equal in both groups.

In principle our results agree with those of Korteweg and Thomas (1939) who compared the reaction of intact and of hypophysectomized mice to benzopyrene dropped on to the skin. In the former papillomas developed in all animals 44–100 days after treatment was started, but in the latter the first papilloma appeared at the 108th day. Only in 14 of 18 hypophysectomised animals surviving for more than 168 days benign tumours developed and in 10 of them they became malignant. These results were obtained in animals in which both groups were treated with the same dose of the carcinogen and in which the administration of benzopyrene was continued for up to 26 weeks. The mice were $F_1$ hybrids the father of which was invariably a dilute brown (Murray Little strain).

The results of Korteweg and Thomas (1939) and our own fit the hypothesis that hormones are the cause of the difference in the response of normal mice on the one hand and pituitary dwarfs or hypophysectomized mice on the other. They indicate also that the role of hormonal factors in the pathogenesis of neoplasms derived from the epithelial cells of the skin is limited to the early phase of tumour development. Progression to invasive or metastasizing carcinomas seems independent of the endocrine status of the host.

### SUMMARY

The susceptibility of the skin of pituitary dwarf mice to the action of DMBA was compared with that of their phenotypically normal litter mates.

Tumours developed in 100 per cent of the controls but only in 60 per cent of the dwarfs even when both groups were treated with equal amounts.

The latent period was always longer in the dwarfs.

This investigation was supported by a generous grant made to the Hugh Adam Department by the McLelland Trust which is gratefully acknowledged.

We should like to express our thanks to Mrs. E. Muir and Miss E. Johnson for valuable assistance.

### REFERENCES

Bickis, I., Estwick, R. R. and Campbell, J. S.—(1956) *Cancer*, **9**, 763.

Della Porta G., Terracini, B., Dammert, K. and Shubik, P.—(1960) *J. nat. Cancer Inst.*, **25**, 573.

Kaplan, H. S., Nagareda, C. S. and Brown, M. B.—(1954) *Recent Progr. Hormone Res.*, **10**, 293.

Korteweg, R. and Thomas, F.—(1939) *Amer. J. Cancer*, **37**, 36.